# How to communicate with patients in written asynchronous online conversations: an intervention study with undergraduate medical students in a cross-over design

**DOI:** 10.3389/fmed.2023.1026096

**Published:** 2023-05-18

**Authors:** Teresa Festl-Wietek, Rebecca Erschens, Jan Griewatz, Stephan Zipfel, Anne Herrmann-Werner

**Affiliations:** ^1^TIME–Tübingen Institute for Medical Education, University of Tuebingen, Tuebingen, Germany; ^2^Department of Internal Medicine VI, Psychosomatic Medicine and Psychotherapy, University Hospital Tuebingen, Tuebingen, Germany; ^3^Deanery of Students’ Affairs, Faculty of Medicine, Eberhard-Karls University of Tuebingen, Tuebingen, Germany

**Keywords:** online communication, written communication, medical students, patient-physician communication, digital communication

## Abstract

**Introduction:**

The patient-physician encounter is the core element in the treatment of patients and the diagnosis of disease. In these times of digitalization, patient-physician communication is increasingly taking place online: patients embrace new possibilities offered digitally, and physicians are encouraged to adapt accordingly. Since a huge part of online communication is written, this study aims to investigate how medical students communicate with patients online by focusing on their written competencies and whether an intervention might improve their competencies.

**Methods:**

This study was performed in an explanatory cross-sectional manner with a cross-over design. Second-year medical students participated. An intervention was developed on how to formulate an appropriate written response to a patient’s request and integrated a longitudinal communication class. The intervention consists of education on general set-up (e.g., greetings), syntax, spelling, content and kind of communication (e.g., appreciative attitude). After meeting a patient in a simulated role play medical students received the patient’s request via a digital platform. The control group had the same simulated role play and the same task but they received the intervention on communication afterwards. Intervention and control group were statistically compared based on a checklist.

**Results:**

Twenty-nine medical students took part in the study. The results showed that the medical students had basic competencies in dealing with written communication independent if they received the intervention (CG: M = 3.86 ± 1.23 vs. IG: M = 4.07 ± 1.03; *p* = 0.625). Similar results were also for the emotional competency ratings (M_CG_ = 3.36 ± 1.08; M_IG_ = 3.67 ± 0.98; *p* = 0.425).The intervention was able to lead to a more appreciative response toward patient.

**Discussion:**

Intervention on basic competencies such as simple language and clear presentation might not be needed as an integral part in medical education. However, medical students should learn how to present empathic and authentic behavior in written online communication.

## 1. Introduction

The patient-physician encounter is the most common task performed by physicians, and it delivers relevant information for diagnosis and treatment of disease (1, 2). Patient-physician encounters still take place in traditional face-to-face interviews. Digitalization is challenging this traditional method of patient-physician encounter. According to the digital law of patient-centered medicine, physicians shall communicate with patients via telemedicine and prescribe online health tools for them (3). Simultaneously, patients more and more often use the Internet for medical research and actively participate in medical forums (4–7). They prefer convenience and anonymity when consulting a doctor on the Internet (6). Vennik et al. (7) found several reasons why patients use medical forums to communicate with doctors: they use them for medical activities like gathering information or staying informed, as well as emotional activities like gaining recognition or expressing emotions and thoughts. Vennik et al. (7) further suggested that online patient-physician communities should be established to help patients with their diseases and to check information delivered online for accuracy and reliability. The emotional activities reported by patients seem to be similar to Roger’s principles for general patient-physician communication: being appreciated, being empathic, and being authentic (8). Basic theory models like Watzlawick and Schulz von Thun should also be considered and implemented in online patient-physician communication (9, 10).

Regarding basic communication models like Watzlawick and Thun, the authors strengthen the relevance of non-verbal aspects in human interaction (9, 10). When chatting in forums, non-verbal aspects might get lost as the people cannot see each other. This implies that the content of the sender’s message becomes more important to the receiver.

In general, patient-physician communication already plays a relevant role in medical training. Efficient patient-physician communication may lead to an improved relationship between patient and physician (11–15). Further, it contributes to more satisfaction for both patients and physicians, as well as higher compliance and better medical outcomes (11–15).

Due to the pandemic of Covid-19, there was a rapid change in online patient-physician communication (16). Patients and physicians had to communicate online by using webcam-enabled computers or smartphones and consultations took place via telephone-conferencing (17, 18). Telemedicine and eHealth presented a high potential for bringing patients and physicians together (19, 20). However, little is known regarding how to best communicate with patients online in a written way (7, 21). For efficient online communication, it is first of all necessary to write a message in an understandable way. Thus, we developed an intervention for medical students on how to write an understandable answer to a patient’s request. We tested this intervention in a cross-over design using a digital platform.

### 1.1. Aim of the study

The aim of the study was to test an intervention on using understandable language in an online patient-physician encounter. This study further aimed to investigate how medical students communicate with patients in writing. The following hypotheses were investigated:

- The intervention group (IG) uses significantly more simple syntax than the controlled group (CG).
- The IG uses significantly more everyday language and the same choice of words and avoided abbreviation and medical jargon in comparison to the CG.
- The IG presents only one piece of information per sentence.
- The IG indicates evidence-based methods.
- The CG uses significantly more emoji than the IG.
- The IG receives significantly higher ratings than the CG in the items of global rating.

## 2. Methods

### 2.1. Study design and participants

This study was performed in an explanatory cross-sectional manner with a cross-over design at the Medical Faculty in Tuebingen, Germany. Second-year medical students were recruited from their curricular teaching on taking medical histories in summer semester 2019 and data were collected during the same period. Medical students were taught by experienced physicians. Participation in teaching was mandatory, but participation in the study was on a voluntary basis. To participate in the study, medical students had to fulfill the following criteria: being 18 years or older, proficient in German language, being in the second year of medical training, having no experiences in written online communication with patients. Thus, we tried to address potential sources of bias.

### 2.2. Longitudinal communication course

The intervention was implemented in an already existing longitudinal communication course on taking medical histories. This communication course is based on basic theory models (8–10) and consists of eight modules. The first two teaching modules focused on providing information, while modules three to eight presented practical units in which the medical students took over the role of physician (please see Table 1 for details). All modules took place at intervals of one week. The data collection was finished after attending module 2.

**Table 1:** Overview of the longitudinal communication class in the second preclinical year.

Teaching module	Kind of teaching	Number of students	Duration	Content	Exercises/role plays
1	Lecture	about 180 medical students	90 min	Basic communication models (Thun, Watzlawick) Basic structure of medical history	Physician with simulated patient
2	Seminar	max. 30 medical students	90 min	Specific patient case Intervention How to give feedback	Patient’s request via digital platform Physician with simulated patient
3	Practical unit	max. 10 medical students	90 min	Taking medical history with a cooperating vs. non-cooperating patient	Medical students take over the role of patient and physician
4	Practical unit	max. 10 medical students	90 min	Taking medical history and simulated ward round	Medical students take over the role of patient and physician
5	Practical unit	max. 10 medical students	90 min	Taking medical history and assessment of psychosocial aspects	Medical student and simulated patient
6	Practical unit	max. 10 medical students	90 min	Taking medical history and taking sexual history	Medical student and simulated patient
7	Practical unit	max. 5 medical students per group	90 min	Taking medical history	Medical student and real patient
8	Practical unit	max. 5 medical students per group	90 min	Taking medical history	Medical student and real patient

### 2.3. Intervention and digital platform

The intervention was implemented in module 2 of the teaching curriculum and lasted around 20 min. The medical students were taught how to communicate with patients in an understandable way by receiving the theoretical input via PowerPoint. The content of this intervention was based on literature-derived research (7, 22–24) containing the following points: (1) general set-up, (2) syntax, (3) content, (4) spelling, (5) scientific procedure, and (6) kind of communication. Please see Table 2 for more details.

**Table 2:** Overview of the checklist.

Headings	Items	Scale
General set-up	Greeting	Yes/No
	Saying goodbye	Yes/No
Syntax	Direct speech	Yes/No
	Simple syntax	Yes/No
	Avoiding metaphors/ passive sentences	Yes/No
	Clear presentation	Yes/No
Context	Using simple and same words	Yes/No
	Avoiding medical jargons	Yes/No
	Avoiding abbreviations	Yes/No
	Delivering one information per sentence	Yes/No
Spelling	More than three errors	Yes/No
	Number of errors	In numbers
Scientific procedure	Presenting evidence-based methods	Yes/No
Asking for medical history information	Asking for allergies	Yes/No
	Reporting on adverse effects	Yes/No
	Reporting medical consequences	Yes/No
Kind of communication	Use of emojis	Yes/No
Showing appreciative attitude	Offering personal call	Yes/No
	Offering contact when having questions	Yes/No
	Asking for fears and concerns	Yes/No
Global ratings	Overall impression	0 (not at all) to 6 (very)
	Scientific competency (e.g., using statistical values, significance)	0 (not at all) to 6 (very)
	Emotional competency (e.g., encouraging patient)	0 (not at all) to 6 (very)
	Adequate text length	0 (not at all) to 6 (very)

The medical students received a case report—using a digital contact form on the GP’s website—involving a patient who consulted her physician due to persistent pain in the lumbar spine. The patient wanted further information on the peridural injection treatment that she had discussed earlier with her GP. The medical students took over the role of clinical clerk to formulate an answer to the patient’s request via a digital platform, which was similar to an online forum and tested in previous studies (25, 26). The online forum was closed and students could enter it only by logins that were provided by the teacher. The login data was anonymous and was not referable to the students by the teacher. Previously, the forum was only used in the medical education setting. The intervention and the task of the online forum were part of the regular course.

As the medical students were in their second year of medical study, they received information on peridural injection and were allowed to use the Internet for further input. We would like to emphasize that they were not expected to find the correct procedure for peridural injection, but they were expected to formulate an understandable answer.

### 2.4. Setting

**Figure 1:**
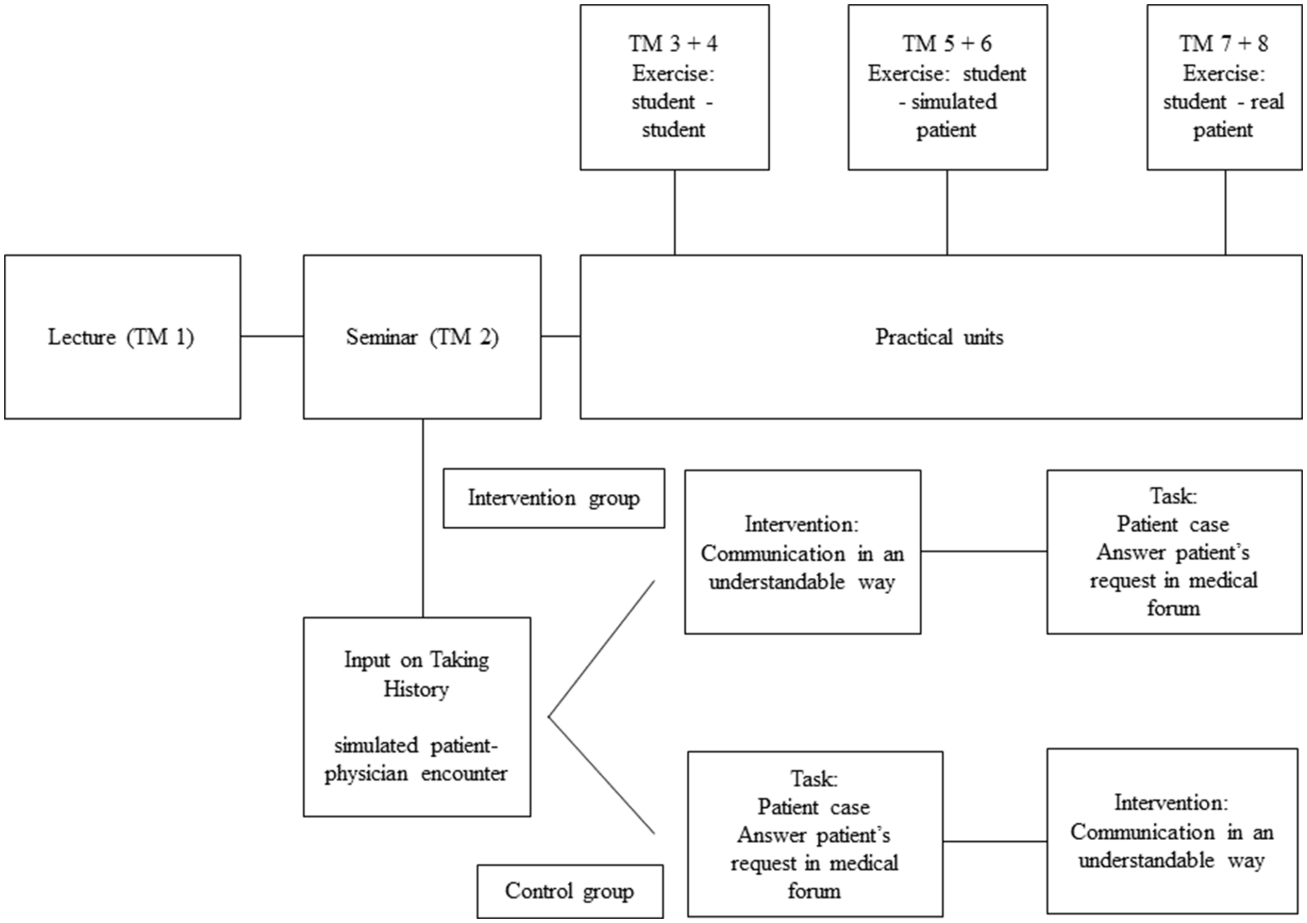
Study design. TM, Teaching module.

The intervention took place in module two of the longitudinal communication class. The class was separated into two groups including intervention and control group. The students were randomized to the intervention and control group. The control group were to directly answer the patient’s request. The intervention group received the intervention first, and then they answered the patient’s request (see Figure 1).

### 2.5. Ethics

The study received ethical approval from the Ethics Committee of the Tuebingen Medical Faculty (443/2018BO2). All participants gave their written informed consent, and all answers were anonymous. So, it was not possible to identify which student took part and who declined. They did not receive reimbursement for their participation. The digital platform was exclusively connected to the university’s Internet.

### 2.6. Data analysis

#### 2.6.1. Checklist

In accordance with the previously described procedure, we aimed to develop a checklist including dichotomous categorical variables (e.g., greetings done or not done) in order to guarantee standardized analysis and evaluation of the medical students’ answers. The checklist was based on study results found by Bientzle et al. (23) and on the Therapeutic Health Concepts Scale (22). The checklist consisted of the following variables: greetings, syntax (e.g., direct address of patient), context (e.g., using same words, no synonyms), grammar (e.g., misspellings), scientific procedure (e.g., diagnostic steps), and kind of communication (e.g., appreciative attitude). The checklist was only available for the reviewers and not for the participating students. Finally, we checked whether the medical students finished their answers. The data was analyzed by two independent reviewers (KE and VD) using the checklist with practical examples. The raters were experts in communication and received a training beforehand. In the overall rating, the reviewer also rated general impression, scientific method, emotionality, and appropriate text length on a 7-point Likert scale from 0 (not at all) to 6 (very). Please see Table 2 for an overview.

#### 2.6.2. Statistics

Statistical analysis was performed using SPSS 25 (SPSS Incorporated, Chicago, IL). The calculated sample size was *n* = 17 per group (power 0.8; effect size 0.5; level of significance 0.05). Data was normally distributed by using the Kolmogorov–Smirnov test. Mean values (M), associated standard deviations (SD), frequencies, and percentages were calculated. In order to test possible differences between the groups, chi-squared tests and t-tests for independent samples were used. The level of *p* < 0.05 was reported as significant. If there were no significant differences between the CG and IG, we reported the results together and presented the correct value of p. Interrater reliability was calculated based on the Intra-Class-Correlation-Coefficient (ICC).

## 3. Results

### 3.1. Demographics

Twenty-nine medical students took part in the study: 59.2% were female, and the average age was 20.7 ± 3.3. Fourteen medical students (48.3%) were in the control group (CG), and fifteen medical students (51.7%) were in the intervention group (IG). Due to technical issues we had to exclude three participants from the control group and two from the intervention group. Interrater reliability measured with ICC coefficient was satisfactory with 0.79.

### 3.2. General set-up

Independent of group, all medical students (*N* = 29, 100.0%) welcomed the patient in their replies. Six students (20.7%) did not complete the task in the prescribed time. When completing the task, all medical students said goodbye to the patients in their replies (*N* = 23, 79.3%). There was no significant difference between the groups (*p* = 0.311).

### 3.3. Syntax

All medical students (*N* = 29, 100.0%) used direct speech, avoided metaphors, and built up information logically in their replies. More medical students in the CG (*N* = 10, 71.4%) used simple syntax in comparison to the IG (*N* = 6, 40.0%). However, the difference was not significant (*p* = 0.089). Thirteen medical students (44.8%) from both groups avoided passive sentences (*p* = 0.588), and 19 (65.5%) presented their replies clearly (*p* = 0.359).

### 3.4. Content

Seventeen medical students formulated their replies in everyday language (58.6%, *p* = 0.550), and most of them (*N* = 23, 79.3%, *p* = 0.311) used the same words in order to receive an understandable answer. Eighteen medical students (62.1%, *p* = 0.597) avoided medical jargon. All medical students in the CG (*N* = 14, 100.0%) avoided abbreviations, while nine (60.0%) medical students in the IG avoided them (*p* < 0.01). Similar results were shown for presenting information (*p* < 0.05): more medical students in the CG (*N* = 11, 78.6%) presented only one piece of information per sentence compared to the IG (*N* = 5, 33.3%).

### 3.5. Spelling

Most of the medical students formulated error-free responses regarding spelling (*N* = 24, 82.8%, *p* = 0.564).

### 3.6. Scientific procedure

Independent of group, most of the medical students (*N* = 26, 89.7%, *p* = 0.501) did not inform the patient about evidence-based methods or ask first for medical history information (*N* = 17, 58.6%, *p* = 0.550). However, most of the medical students in both groups (*N* = 24, 82.8%, *p* = 0.164) reported in their replies on adverse effects and medical consequences.

### 3.7. Kind of communication

None of the medical students used an informal communication like any of the emojis that were offered in the medical forum to help communicate with the patient. Further, there was a significant difference in appreciative attitude (*p* < 0.05). Most of the medical students in the IG (*N* = 13, 86.7%) showed an appreciative attitude in their replies, like being available for questions or offering a personal call, while only 7 (50.0%) medical students in the CG fulfilled this aspect.

### 3.8. Global rating

In the global rating, there was no significant difference between the CG (M = 3.86 ± 1.23) and the IG (M = 4.07 ± 1.03; *p* = 0.625). Similar results were also shown for the scientific (M_CG_ = 3.86 ± 0.95; M_IG_ = 3.87 ± 0.64; *p* = 0.975) and for the emotional competency ratings (M_CG_ = 3.36 ± 1.08; M_IG_ = 3.67 ± 0.98; *p* = 0.425). The highest results were achieved regarding rating of adequate text length (M_CG_ = 4.79 ± 1.48; M_IG_ = 4.93 ± 1.49). There was again no significant difference between the groups (*p* = 0.971).

## 4. Discussion

This study aims to examine an intervention on using understandable language in an online patient-physician encounter via a digital platform. Furthermore, it investigates how medical students communicate with patients in writing In general, the results indicate that the intervention did not increase the medical students’ knowledge as expected. Further, the results indicate that the medical students show a basic competency in professionally communicating with patients in writing. Independent of the intervention, medical students welcomed and said goodbye in their replies to the patients. They also used appropriate language and syntax and created error-free responses. According to the results, the intervention could deliver an appreciative attitude in online patient-physician encounters. Further, no one used an informal way like emojis to communicate with the patients in an informal way. This indicates the professional attitude of the medical students when dealing with patients online. In the following, we discuss the outcomes of our intervention.

### 4.1. Professional attitude

One possible reason for the outcome of the intervention could be the professional attitude of medical students. From the beginning, medical schools support professional identity formation, and medical students are aware of their role and see themselves as part of the medical profession (27, 28). In the course of their intervention, the medical students seemed to adopt a professional attitude toward the patients and appropriately answered their questions. As they got to know the patients beforehand in simulated role play, their professional attitudes might have been strengthened.

In general, studies could show that medical students possess important skills and values such as altruism and communication when entering medical school (24, 25). However, as empathy and patient-centered communication skills tend to decline during the course of medical school, it might be helpful to implement such a course, not in year two, but rather later in the clinical years (29). Regarding the results, almost all medical students in the IG showed an appreciative attitude in their replies, while it did not indicate that this intervention could foster empathic behavior.

### 4.2. Digital skills of medical students

The myth of the digital natives is the subject of an ongoing discussion, and one should be careful in assuming that this generation can easily transfer personal digital competencies into professional ones (30, 31), although several studies confirm that students have digital competencies (32–34). Bientzle et al. (23) showed that medical students were able to communicate appropriately with patients in an online forum. This study indicates that medical students already possess basic competency in writing answers to patients’ requests. Further, medical students possess digital skills when looking for information online (33). In this study, they showed their digital skills in finding reliable information on adverse effects of peridural injection and reported them in their reply to the patient.

### 4.3. Online communication as a challenge

However, one should not forget that online communication presents an ongoing challenge. On one hand, medical forums are very popular with patients (4–6). They use these forums to remain anonymous, especially when it comes to shameful topics or when it is difficult to find time to visit a physician (6). On the other hand, relevant aspects of communication such as the non-verbal get lost (9, 10). Furthermore, general principles for patient-physician communication such as empathy and authenticity are harder to convey online (8). This study showed that medical students have basic competencies and are able to communicate appreciably with patients in writing. Future studies should focus on how to deliver the best non-verbal aspects like authenticity and empathy in written online patient-physician communication, as patients also prefer written communication (6).

Furthermore, the written communication presents a challenge in delivering technically correct answers. Medical students did not inform the patient about evidence-based methods in their reply. Bientzle et al. (23) suggested that medical students could find it difficult to inform patients in a written way as they are afraid of being held to account on this information. The digital platform used in this study presents a valuable tool in order to investigate online communication in future studies.

### 4.4. Strengths and limitations

The strength of this study includes the investigation of medical students’ communication skills via a digital platform in a cross-sectional design. This study, further, reveals a professional attitude of medical students in an online communication setting. Our intervention was limited. It only contributes to a more appreciative attitude in written online communication. Possible reasons for the failure of our intervention were discussed above. Moreover, the intervention only lasted 20 min which might be too short to affect the students’ competencies. When regarding methodical aspects most items were assessed as dichotomous variables that influenced the significance of the results found. The items of the checklist should be assessed on a multivariate scale in further studies. Furthermore, due to technical issues our calculated sample size was not attained but the data were normally distributed. A further potential limitation of this study is that the generalizability of the findings must be handled with care. Here, the medical students showed basic competencies in written online communication. However, it cannot be expected that all medical students automatically have these competencies, so individually adapted teaching might be necessary. Furthermore, we did not ask for the students’ socio-cultural background and their linguistic abilities which should be considered in future studies.

## 5. Conclusion and further directions

This study showed that medical students possess the basic competencies needed to write an appropriate answer to a patient’s online request. Further interventions on simple language or syntax might not be needed as an integral part of the medical curriculum. However, one should consider that relevant non-verbal aspects like empathy and authenticity could get lost in written online patient-physician communication. As written online communication seems to be popular with patients, future studies could focus on how to best integrate these non-verbal aspects in this kind of communication as relevant aspects of basic communication models should be implemented in each kind of medical training of patient-physician communication (8–10).

## Data availability statement

The raw data supporting the conclusions of this article will be made available by the authors upon reasonable request, subject to data protection requirements.

## Ethics statement

The studies involving human participants were reviewed and approved by the Ethics Committee of the Tuebingen Medical Faculty (443/2018BO2). The patients/participants provided their written informed consent to participate in this study.

## Author contributions

AH-W and TF-W were responsible for the design and conduction the study, as well as acquisition, analysis, and interpretation of data. AH-W and TF-W drafted the first version of the manuscript. RE and JG were involved in data analyses, interpretation, and revised the manuscript critically. SZ made substantial contributions to the study design and revised the manuscript critically. All authors approved the final version of the manuscript and agreed to be accountable for all aspects of the work.

## Funding

The authors acknowledge support with financing publication fees by Deutsche Forschungsgemeinschaft and Open Access Publishing Fund of the University of Tuebingen.

## Conflict of interest

The authors declare that the research was conducted in the absence of any commercial or financial relationships that could be construed as a potential conflict of interest.

## Publisher’s note

All claims expressed in this article are solely those of the authors and do not necessarily represent those of their affiliated organizations, or those of the publisher, the editors and the reviewers. Any product that may be evaluated in this article, or claim that may be made by its manufacturer, is not guaranteed or endorsed by the publisher.
